# Changing streetlighting schemes and the ecological availability of darkness

**DOI:** 10.1098/rsif.2023.0555

**Published:** 2024-02-28

**Authors:** Sam Morrell, Jennifer Hatchell, Freddy Wordingham, Jonathan Bennie, Maisy J. Inston, Kevin J. Gaston

**Affiliations:** ^1^ Environment and Sustainability Institute, University of Exeter, Penryn, Cornwall TR10 9FE, UK; ^2^ Centre for Geography and Environmental Science, University of Exeter, Penryn, Cornwall TR10 9FE, UK; ^3^ Department of Physics and Astronomy, University of Exeter, Stocker Road, Exeter, EX4 4QL, UK

**Keywords:** ecology, artificial light at night, Monte Carlo radiative transfer, light pollution

## Abstract

Artificial light at night (ALAN), from streetlights and other sources, has a wide variety of impacts on the natural environment. A significant challenge remains, however, to predict at intermediate spatial extents (e.g. across a city) the ALAN that organisms experience under different lighting regimes. Here we use Monte Carlo radiative Transfer to model the three-dimensional lighting environment at, and just above, ground level, on the spatial scales at which animals and humans experience it. We show how this technique can be used to model a suite of both real and hypothetical lighting environments, mimicking the transition of public infrastructure between different lighting technologies. We then demonstrate how the behaviour of animals experiencing these simulated lighting environments can be emulated to probe the availability of darkness, and dark corridors, within them. Our simulations show that no single lighting technology provides an unmitigated alleviation of negative impacts within urban environments, and that holistic treatments of entire lighting environments should be employed when understanding how animals use and traverse them.

## 1. Introduction

Biological impacts of artificial light at night (ALAN), from streetlights and other sources, have long been recognized (e.g. [1–4]). However, recent years have seen a dramatic increase in published empirical studies (reviewed in [5–7]). These have demonstrated that the kinds of ALAN illuminance that many wild organisms may encounter can have important impacts on individuals (including physiology and behaviour), populations (including reproduction and mortality), communities (including structure and composition) and ecosystems (including function and process). Such impacts span microbes, fungi, plants and animals, and occur across terrestrial, freshwater and marine realms [5,7–9]. These impacts are widespread, because ALAN itself is distributed extremely widely and is continuing to increase both in local intensity and extent [10–13], and because the forms of ALAN that are being emitted are becoming biologically more problematic (particularly having greater blue emissions; [14]).

An obvious approach to mitigating the biological impacts of ALAN is through the adequate provision of dark or darker corridors, by which animals can move at night while experiencing reduced artificial lighting [15]. This requires understanding of spatial patterns in the occurrence of ALAN. Most research on this distribution has been done using data from sensors on board satellite platforms (particularly DMSP/OLS and SNPP VIIRS-DNB; [16,17]). However, while the spatial extent of these data is large, their spatial resolution is quite coarse (DMSP–OLS, 2.7 km; VIIRS, 740 m) relative to that at which many organisms are making behavioural decisions. The situation is improving as data from new satellite platforms become available (e.g. [18–20]), but variation in the angles to the zenith at which these are obtained can be analytically complex to address, and the data are often expensive to obtain for research purposes. The cost of aerial night-time imagery from plane or drone platforms is also often prohibitive, requiring dedicated acquisition flights (but see [21,22]). Flights using such platforms are also frequently restricted both at night, and over built-up areas. A further limitation common to all of these approaches is that they consider ALAN in two dimensions (2D), although animals will respond to its three-dimensional (3D) occurrence.

An alternative, and potentially generalizable, approach to obtaining fine resolution data on the spatial occurrence of ALAN is to build models from the ‘bottom up', seeking to simulate its distribution from the emissions of the lamps that are present. This requires determining the concentrations and spectral characteristics of the photons propagating through each point in the studied volume. Computational methods to account thoroughly for these effects have not been forthcoming, meaning that it has been the norm to consider only the effects of individual lighting fixtures in isolation. A method is required that can simulate the entire environment.

Bennie *et al*. [23] apply a computational method to model the ground-level spatial distribution of light in an urban environment and demonstrate how this can be used to probe the dark corridors within the simulated area. However, this work treats lights as point sources which emit isotropically in their lower hemisphere, losing the nuanced spatial information resulting from the diverse use of different luminaires. Furthermore, this method is only 2.5D, in that the lights and environment themselves are treated in 3D, but the output is a 2D plane at ground level. This method also only provides a first-order approximation, considering only direct emissions from light sources, but ignoring scattering, absorption and reflections.

There are a number of commercial software products available for both interior and exterior lighting design that overcome the first of these issues, such as AGi32, DIALux and Relux, as they incorporate treatments of light intensity distributions. However, they too tend to treat only brightness upon surfaces, treat light in an anthropocentric manner (working in terms of photometric as opposed to radiometric quantities) and tend to pitch their toolsets at lighting engineers.

Meanwhile, where some of the key scenarios and observables important for studies of ALAN are not served by these off-the-shelf tools, researchers have developed their own codes. Their approaches range from analytical geometric treatments of radiative transfer, such as Kocifaj [24], to Monte Carlo methods, such as ILLUMINA [25–28] and the work of Kolláth & Kránicz [29]. However, owing to much of the work around ALAN stemming from astronomy, these tools are often optimized for studies of skyglow, and hence designed for atmospheric radiative transfer. Truly to probe the availability of darkness to organisms traversing changing lighting environments, we require a generalizable model that can account for both the spatial and spectral variation of light from different luminaires at ground level across the studied environment. In this work, we present such a model. We will first introduce the theory underlying it, then lay out our testing and validation methodology, before progressing toward a specific worked example of its application in an ecological context.

## 2. Methods

Our model is rooted in the Monte Carlo radiative transfer (MCRT) method. Given knowledge of the luminaires present in the studied area, MCRT allows simulation of the spatial and spectral distribution of photons as they appear in the real environment. Owing to its computational nature, this technique also allows alternative lighting schemes to be posited and the lighting consequences simulated within the same pipeline. With the resulting simulated lighting environments, we then employ, as a worked example, a weighted random walk algorithm to simulate the response of an arbitrary organism to this intensity field. We use this technique to probe for the availability of darkness and dark corridors.

### 2.1. Monte Carlo radiative transfer

At their core, all radiation transfer solvers attempt to solve a single differential equation, known as the equation of radiative transfer,

2.1 $$\frac{dI_{\nu }}{d\tau }=S_{\nu }-I_{\nu \;},$$

which describes how the specific intensity of light *I_ν_* at a given frequency *ν* changes as a function of optical depth *τ* in a given medium. *S_ν_* is the source function, which incorporates position-dependent treatments of absorption, emission and scattering within the material. An analytical solution to (2.1) is forthcoming for a beam of light at a single frequency. However, in all but the most trivial real-world cases, particularly where spatial and spectral information about the light field are scientifically interesting, analytical solutions become intractable. Furthermore, radiative transport problems are inherently nonlinear, in that very small perturbations in input parameters and environment can result in disproportionate changes in outputs. Hence, even for a simplistic system, the innumerable number of possible interactions between photons and the materials make solving the intensity field through traditional means impossible—an alternative approach is required.

Instead of attempting to solve the radiative intensity field in a single complicated, many-dimensional integration, we may instead consider the propagation of the individual photons which comprise it. The Monte Carlo method applied to radiative transfer, known as Monte Carlo radiative transfer (MCRT), simulates the lives of myriad packets of photons propagating throughout a physical system. Each packet's movements and interactions are determined through random sampling of physically motivated probability density functions (PDFs), each representing some aspect of the photon packets' interactions with their environment. Sufficient sampling yields a reproducible, convergent result which describes the state of the radiation field throughout the modelled system. The elegance of the MCRT method is that few assumptions are required to achieve a convergent result. Increasing the number of sampling photon packets over which the simulation is discretized can be thought of as increasing its precision, hence improving the signal-to-noise ratio (SNR). Furthermore, increasing the fidelity and accuracy of both the geometry and PDFs improves the accuracy of the simulation. Thus, provided with absolute knowledge of the structure of the environment and number of photon packets present in real life, MCRT simulations would converge upon reality.

Astrophysics has made extensive use of MCRT methods (for early application e.g. [30,31]), owing to the field's reliance upon light as the sole source of information about studied physical systems. MCRT methods have also seen wide use in medicine, as the technique is safely able to estimate the radiation dose deposited in bodily tissues during nuclear medical treatments and diagnostic radiography [32]. The predecessor to our MCRT solver originated in biophysics, having been applied to photodynamic therapy (PDT) of non-melanoma skin cancer [33] and the detection of breast cancer [34].

In this work, we adapt the design of Wordingham [35], which reflects many of the abstract concepts presented in Harries *et al*. [36], to create an MCRT tool for use in studying ALAN. Herein we describe the MCRT method itself and how we have tailored its implementation for use in studying ALAN by following the hypothetical lifetime of a photon packet as it propagates through a simulation.

#### 2.1.1. Emission

To simulate the emission of a photon packet from any light source, two intrinsic properties must first be specified: its direction of emission and photon energy, the latter of which determines the frequency ν and wavelength λ of the photons within the packet. The total luminosity of an emissive source *L*, also known as radiant flux, over a unit time Δ*t* is divided between *N* sampling photon packets such that each packet contains energy *ɛ*, expressed as

2.2 $$\frac{\epsilon }{\Delta t}=\frac{L}{N}.$$

The initial wavelength of the photons within the packets, sampled from a cumulative distribution function (CDF) created from the light source's emission spectrum, determines the wavelength-dependent optical properties of the medium into which it is emitted. We reiterate that all photon packets emitted from a given light source have the same energy, as prescribed in (2.2). Thus, the wavelength assigned to a given photon packet essentially scales the number of photons contained within it.

We simulate the non-isotropic emission characteristic of real luminaires by sampling randomly from their manufacturer-provided photometry. The measured luminous intensities *C_ϕ_*_,*θ*_, provided in units of candela, that constitute the photometric web are arranged in a spherical shell made up of vertical planes, which are oriented as a function of azimuthal angle *θ* around the polar axis. We create CDFs from each of the planes by normalizing the intensities by the total intensity of the plane. The azimuthal CDF is then created by normalizing the intensity of each plane by the total luminous flux of the light intensity distribution (LID). To emit a photon packet, we first sample from the azimuthal CDF to determine its heading *θ*. The polar angle *ϕ* is then determined by sampling from the CDF of the plane whose angle is nearest the drawn *θ*. The final emission position and orientation of the packet within the simulation are then found by subjecting it to the same translational and rotational transformation as the emitting light source.

#### 2.1.2. Propagation

The simulation domain is discretized into a regular three-dimensional arrangement of volumetric pixels, or voxels, over which continuous spatial variables are recorded. Each photon packet propagates through the simulation in steps, with interactions determined by three distance measurements. First, if the distance to the nearest surface *D*_surf_ is the shortest, we handle the interaction via the surface's tagged attribute. Second, if scattering distance *D*_scat_ of the medium through which the photon packet is propagating is the shortest distance, we handle the scattering event as detailed in §2.1.5. Finally, if neither *D*_surf_ or *D*_scat_ are adequately short, we handle the photon packet hitting the boundary of the current voxel in the output grid and transfer it to the neighbouring voxel. A simulated photon packet is always contained within a single voxel in this grid at any one time—packets which reach the domain boundary are pruned from the simulation.

As the surfaces of objects, such as terrain and buildings, are represented by triangle meshes, we determine if a packet will hit a surface, and if so *D*_surf_, by hit-scanning. This involves tracing a ray in the direction of travel of the packet and checking for an intersection with a triangle. The algorithm for performing hit-scans on triangular meshes is comparatively computationally expensive. Hence, to reduce the number of triangles we need to check, we employ a hit-scan octree. This spatially partitions the simulation domain recursively into eight equal volume subdivisions until we meet the desired number of triangles per cell, or the desired limit in refinement.

As a photon packet with energy *ɛ* travels a distance *l* through a voxel over a unit time Δ*t*, it contributes an energy

2.3 $$\Delta E=\epsilon \frac{l}{(c/n_{v})\Delta t},$$

where *n_v_* is the refractive index of the material in which the packet is travelling (*n_v_* = 1.0 for the air in this work), and *c* is the speed of light in a vacuum. From this, the average energy density within the voxel's volume is therefore

2.4 $$U_{E}=\frac{\epsilon }{V}\sum \frac{\;l}{(c\;/\;n_{\nu })\;\Delta t}\;,$$

where *V* is the volume of the voxel over which the contributed energy is averaged. The sum is over fractional lengths over different refractive indices of materials within the voxel. This is essentially the radiant flux that is observed across all directions. We note that this measure is a computational analogue to the measure of volume concentration of anthropogenic photons described in [37], quantifying the energy concentration of photons instead of the number density. We can output the three-dimensional energy density of simulations, as well as synthetic measurements from a single point, such as images or spectra.

#### 2.1.3. Reflection

Our reflection model consists of specular and diffuse components. A specular reflection, seen mostly on shiny surfaces, occurs when incident light is reflected in a single, deterministic direction. A mirror is a near perfect specular reflector. Conversely, photon packets subjected to Lambertian, or diffuse, reflection are reflected in a random direction in their incident hemisphere. Hence, the brightness of a perfect Lambertian reflector is insensitive to the direction in which it is viewed, e.g. an integrating sphere. Most materials exhibit a combination of both components. Hence, we define specularity to specify the proportion of reflections which are specular. A uniform random draw determines to which mode of reflection each photon packet is subjected: the reflection is specular where the random draw is less than the specularity. Realistic materials also preferentially reflect or absorb photons based on their wavelength, hence we also include a spectral reflectance for each component, expressing the probability of reflection as a function of wavelength. A further uniform random draw is compared with the reflectivity at the wavelength of the photon packet, with values larger than the spectral reflectance at that wavelength being killed and pruned.

#### 2.1.4. Material interfaces

Most materials are not completely opaque, exhibiting at least some form of subsurface scattering or translucency. Hence, surfaces can indicate to our MCRT solver that they comprise an interface between two materials. As the photon packet crosses this interface, the stored optical properties for the material the packet is propagating through—which we cache with the photon packet structure itself for computational efficiency—are updated to those of the new medium, the trajectory of the photon packet is adjusted for the change in refractive index, and the packet is allowed to continue to propagate. It is through this mechanism that we can model complex optical effects, such as refraction and subsurface scattering, in optical materials.

#### 2.1.5. Scattering

The scattering coefficient *μ_s_* is inversely proportional to the mean distance a photon packet is likely to travel through the current medium before an elastic scattering event occurs. As a photon packet scatters, it is deflected from its current trajectory by some angle, determined by phase functions that are used to model the anisotropy factor within a given medium. For this work, we adopt a value of *μ_s_* = 1 × 10^−6^ m^−1^ for air, simulating a clear evening with no haze, effectively allowing the photons to travel with no scattering in air. We are currently working on a more thorough scattering treatment, allowing us to model more diverse weather conditions, and longer path lengths, where scattering may be important.

#### 2.1.6. Model validation

We adopted a rigorous validation strategy for our code. First, we used test-driven development, meaning that new features are developed in tandem with accompanying unit tests. These tests currently cover 70% of our codebase, matching industry recommendations. Along with unit-testing components in isolation, we also ran detailed integration test cases to ensure the physicality of our model. Details of our testing strategy and test cases can be found in the accompanying digital electronic supplementary material.

### 2.2. Constructing digital twins of urban environments

To apply our model to an urban environment, we must first create a digital twin. Digital twins represent the key features of a given system which we wish to model in virtual form. In this work, we reproduce digitally the Stoke Hill area of Exeter, UK, including terrain and buildings, represented by geometric triangular meshes, the reflectance characteristics of the surface they represent, and light sources. The terrain mesh was generated using the Environment Agency LiDAR Composite Digital Terrain Model (DTM) 2020 [38] and the Digital Surface Model (DSM) 2020 [39]. First, we generated a two-dimensional terrain mesh by performing a Delaunay triangulation, using the *Triangle* library [40], upon the input footprint of the study plot. We then extruded each of the vertices in this mesh along the *z*-axis to match the height of the DTM at that location.

We formed building geometry by performing a Delaunay triangulation upon the building footprints within the study plot. Building footprints were provided by the Ordnance Survey's VectorMap Local [41] product. We then extruded the mesh along the *z*-axis by the maximum difference between DTM and DSM within the building footprint. A treatment of roof geometry is possible but beyond the scope of this work and should only have a small effect on the ground-level lighting distribution.

Geometric surfaces in the simulation can be tagged with an attribute which dictates how incident photon packets interact with it. This flexibility is one of the key benefits of our modelling approach, as each object can be imparted with an attribute that best represents its properties.

#### 2.2.1. Lighting

We constructed three classes of lamp to represent the street lighting technologies widely in use around the city of Exeter, UK: high-pressure sodium (HPS), ceramic metal halide (CMH) and light emitting diode (LED). We adopted either the input power as specified by Devon County Council (the local authority responsible for street lighting in Exeter), or, for hypothetical lighting scenarios, the mean derived from the lamps present in the study plot. The total luminous flux was then calculated for each lamp from the input power using the mean luminous efficacy found on bulb datasheets. We then converted from luminous to radiometric flux through the luminous efficacy

**Figure 1.**
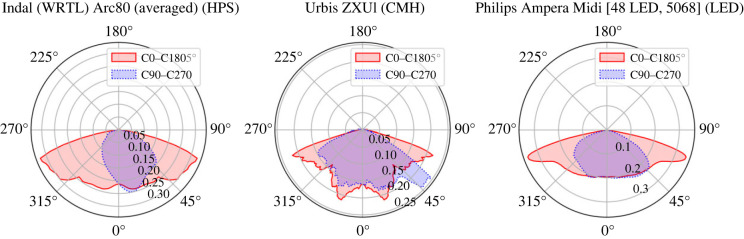
Slices of the two-dimensional PDFs from which we sample the direction of emission for each of the streetlights modelled in the simulation. The C90-C270 plane (blue) shows the distribution as if looking from the left of the lamp, and the C0-C180 plane (red) shows the distribution as if looking from the front. Note that these are derived from the measured light intensity distributions of their real counterparts.

2.5 $$\Phi_{e}=\Phi_{V}\frac{\int_{0}^{\infty }\Phi_{e,\lambda }d\lambda }{\int_{0}^{\infty }V_{M,\lambda }\;\Phi_{e,\lambda }d\lambda },$$

where Φ*_e_* and Φ*_V_* are the radiant and luminous fluxes respectively, Φ*_e_*_,*λ*_ is the (in this case relatively scaled) spectral radiant flux, and *V_M_*_,*λ*_ is the spectral luminous efficiency. We used the modified Judd–Vos photopic luminous efficiency *V_M_*_,*λ*_, which incorporates the Vos [42] corrections to the Judd [43] revision of the CIE 2 degree colour-matching functions [44]. This yields the radiant flux, in watts, that is used for the light sources in our models. The specifications of our representative light sources are detailed in table 1, with the LIDs determined from the lantern of each luminaire shown in figure 1.

**Table 1.** Summary of lighting technologies and specifications used within this simulation.

	high-pressure sodium (HPS)	ceramic metal halide (CMH)	light emitting diode (LED)
lantern	Urbis ZX1	WRTL Arc 80	Urbis Ampera Midi 73W
bulb	GE Lucalox XO [45]/Philips Master SON-T PIA Plus [46]	Philips CPO-T [47,48]	Philips 48 LED Array [49]
input power (W)	70	60	73
nominal luminous efficacy (lm W^−1^)^a^	88	115	139
luminous flux (lm)^a^	6160	6900	10 147
radiant flux (W)^a^	12 381	20 977	21 080

^a^Parameters are estimated from the provided combination of lamp/bulb technology.

**Figure 2.**
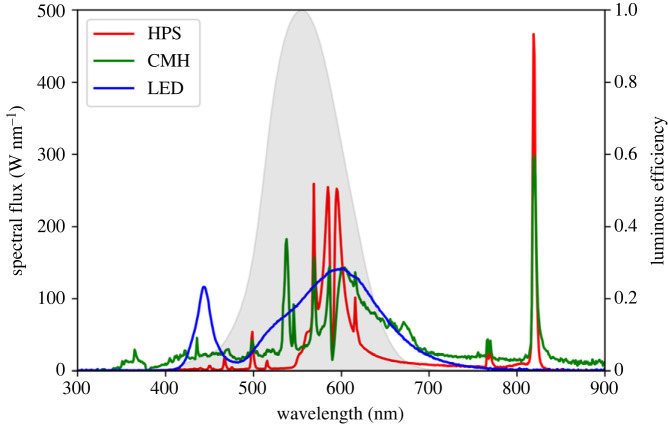
The spectral fluxes adopted for the emission of the HPS, CMH and LED streetlights in this work, shown in red, green and blue, respectively. The spectra represent the PDFs from which the wavelengths of emitted photons are sampled, normalized by the radiant flux of each light. The photopic response of the human eye [44] is overlaid (shaded grey) for reference.

As the emission spectra for each of the provided lamps is not readily available in a machine-readable state, we estimated reasonable emission spectra by matching the technology (HPS, CMH, LED) and correlated-colour temperature (CCT) against a spectrum in the LICA-UCM spectral database [50,51]. These spectra are scaled to match the radiant flux of the simulated luminaire. The final spectral flux used for each lamp type in the simulation is shown in figure 2. For all simulations in this work, we emit 10^6^ photon packets per light source.

### 2.3. Application: dark corridors across urban roadways

**Figure 3.**
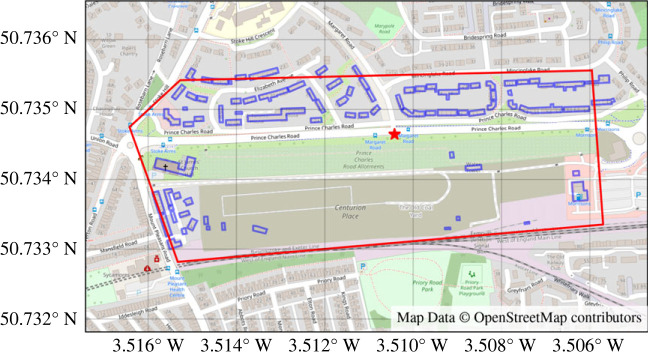
The study area for our simulation (red). The target road is shown running from west to east through the centre of the domain. It is bounded to the north by the suburb of Stoke Hill, and to the south by an area of darkness comprising allotments and disused industrial land. The buildings included in the digital twin are shown as blue polygons. The position of the deactivated lamp in our second tranche of simulations is marked with a red star.

To apply our model, we chose an area around Prince Charles Road in Exeter as our study plot (figure 3). This plot contains a straight road with which we can study the presence of dark corridors. As it is bounded to the north by the ‘Stoke Hill' suburb of Exeter, and to the south by an area of darkness, this is an environment across which we would expect organisms to move at night.

The geometry in the simulation has all been tagged with a composite reflectance model, detailed in §2.1.3, with spectral reflectances taken from version 7 of the USGS spectral library [52]. We set the specularity of materials using the nominal values for equivalent materials in the spectral material database [53,54]. As the modelled light sources occur on roads and footpaths, we model the terrain as tarmac. Assuming dry conditions, we set the specularity of this material to 1%; meaning that most of the reflections are diffuse (Lambertian). For spectral reflectance, we adopt a dark-grey asphalt shingle material (sample ID: GDS367) reminiscent of UK roads. As buildings in the study plot are primarily of red-brick construction, we adopt a medium red brick reflectance (sample ID: GDS353) with a specularity of 0.1%. Both spectral reflectances are plotted in the electronic supplementary material.

A strength of our computational approach is that it allows us to hypothesize alternative lighting scenarios and investigate their impact upon the lighting environment. In this work, we first simulated scenarios where each of the representative lamps detailed in table 1 are installed at regular spacings of 20, 25, 30, 32, 35 and 40 m along roads within the simulation; 32 m is the mean of the actual distance between lamps in this environment. These simulations were intended to probe the availability of darkness and dark corridors through the modelled environment, while keeping the characteristics of the light distribution close to reality. Our second tranche of simulations reproduced these initial scenarios exactly, aside from turning a single lamp off. We strategically chose this lamp to be opposite the road junction leading to Margaret Road (figure 3). We intend this as a proof-of-concept rather than a policy recommendation, as the interruption in lamps along the access road to the north creates an artificial dark corridor to the objective.

We emulated the behaviour of animals traversing the environment by performing weighted random walks across isoheight planes of energy density extracted from each simulation. For each scenario, we ran an ensemble of walks across the simulation domain, where the response of the animal is mimicked by a photophobic walker. Every iteration, the walker first performed a random draw to propose a move to the adjacent pixel in one of the four cardinal directions. Weights were assigned to each direction to adjust the frequency with which they were drawn. Each walker was assigned an energy density threshold. If the pixel in the proposed direction was above this threshold, or the proposed pixel was outside of the simulation domain, the proposed movement was rejected. Otherwise, the walker moved to the proposed pixel. This process repeated either until the walker reached its objective, or the maximum number of iterations was reached, in which case the walk was considered failed. We validated this algorithm following the methodology of McCrea & Whipple [55]. Details of this are included in the electronic supplementary material.

**Figure 4.**
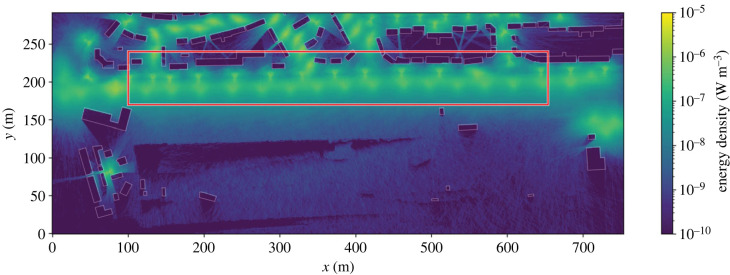
The ground-level energy density outputted from the initial simulation upon our digital twin, using the position, type and power of the lights installed in the actual study plot. Building footprints are outlined in white. The subsection of the grid across which we perform random walks is overlaid in red.

The situation we posed for the random walkers is one encountered regularly by nocturnal organisms in the wild: a road crossing. We had the walkers travel from the dark area in the south, to the housing estate in the north; across the well-lit road that bisects the simulation domain. To avoid the excess darkness present at the edges of the simulation domain, due to missing light sources outside of the study plot, we added a 100 m margin to the eastern and western edges of the domain that the walkers traverse. Having simulated each of our lights in isolation, we found that 100 m was around the median distance from the light source of 10^−10^ W m^−3^ energy density contour at ground level, making this a cautious estimate. To encourage the walkers to cross the walk region, shown in figure 4, we used the weights 0.5, 0.2, 0.1 and 0.2 for the north, east, south and west cardinal directions, respectively. We intuit these weights by assuming the target to be the priority but also allowing lateral and backward movement. Note that these walkers are not meant to mimic the behaviour of specific animals that may be moving through the space, but to act as generic photophobic ones. To sample the full simulated dynamic range, we chose thresholds in log_10_ energy density = −10.0 − −5.0 in steps of 0.5 dex. Given the short 70-pixel distance, we provided walkers a maximum of 1000 iterations to achieve the northern boundary of the domain, else we considered the walk failed. For each permutation of lighting technology and spacing, we allowed 100 000 walkers to traverse the slices of energy density at ground level as well as at 4 m and 10 m above ground level. These heights are intended to correspond roughly to the heights experienced by small mammals, insects and bats, and birds, respectively.

## 3. Results

We first tested our model by simulating the actual luminaires that are installed in the environment, also serving as a validation of the digital twin. Luminaires exhibit the spatial and spectral characteristics of their real-world counterparts, and shadows are cast both by buildings and topography (figure 4), demonstrating that the inputs are correctly representing the environment, and the code is working as expected. These initial simulations showed that the higher luminous efficacy of modern lamps translates to real-world increases in brightness at ground level. The same input power as traditional gas discharge lamps generates around 40% higher radiant flux, translating to an increase in peak photon energy density at ground level of around 12% for LED and 29% for CMH.

**Figure 5.**
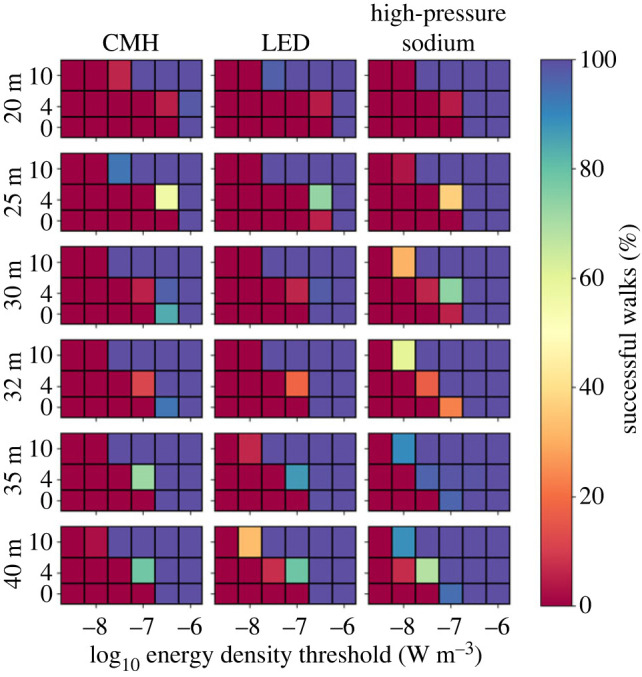
The success rate of the ensembles of random walkers for each scenario we constructed. The rows of the grid show spacing between light sources along the road increasing top-to-bottom. The three columns show results for CMH (left), LED (centre) and HPS (right) lamps. Each panel shows the percentage of walkers that make it completely across the simulation domain at a given energy their energy-density threshold (*x*-axis) and height above the ground (*y*-axis).

**Figure 6.**
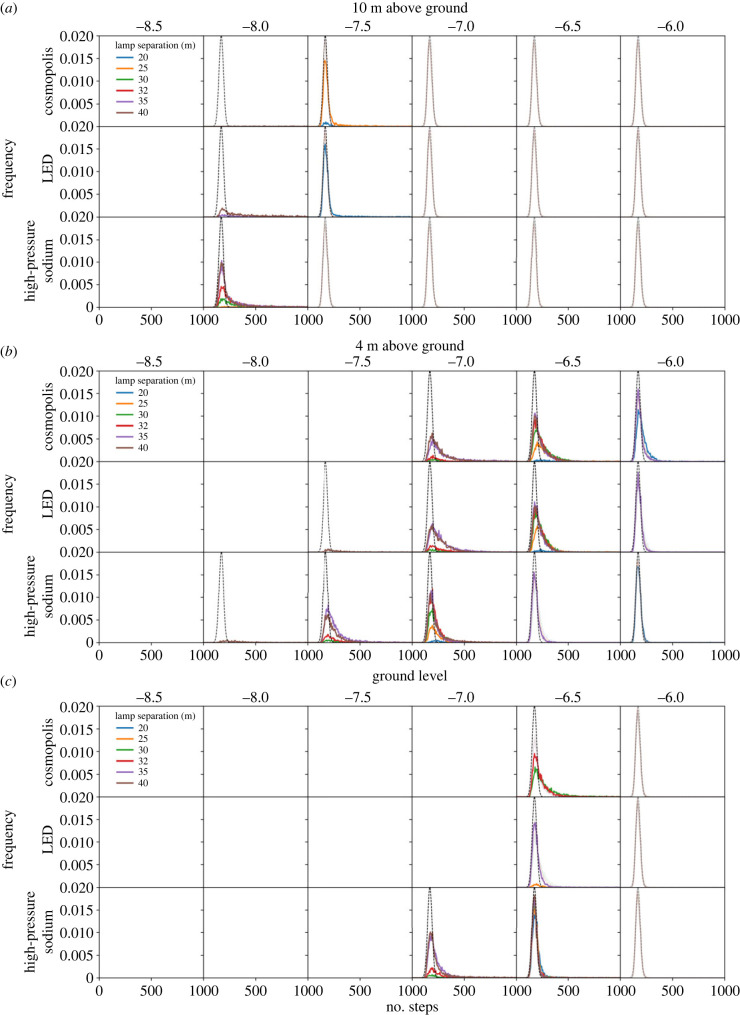
The distributions of random walker steps at 10 m (*a*), 4 m (*b*) and 0 m (*c*) above ground level. The distributions are shown in the grids of panels as a function of representative lighting type (rows), log_10_ energy density threshold above which the proposed move is rejected (columns), and separation between lamps (colours). The nominal distribution is overlaid in each plot as a solid dashed line. No lines indicate no walks were successful. Those distributions for which all walkers are successful are shown faintly for reference.

We then simulated the scenarios described in §2.3. Regardless of light type, height and spacing, a light barrier is formed along the roadway, as indicated by a minimum energy density threshold below which no walks succeed (figure 5). The brightness and spatial consistency of this light barrier is indicated in each scenario by an upper energy density threshold, above which all walks succeed. Between these extremes we see variation across two orders of magnitude in energy density (10^−8.5^ − 10^−6.5^ W m^−3^). To understand these intermediate cases better we plotted the distributions of numbers of steps per walk for scenarios where walks both failed and succeeded (figure 6). For a point of reference, we pooled the distributions where all walks succeed and fitted them with a Gaussian to derive a nominal best-case distribution. Perturbation away from this distribution indicates how a given scenario impedes the path of walkers.

**Figure 7.**
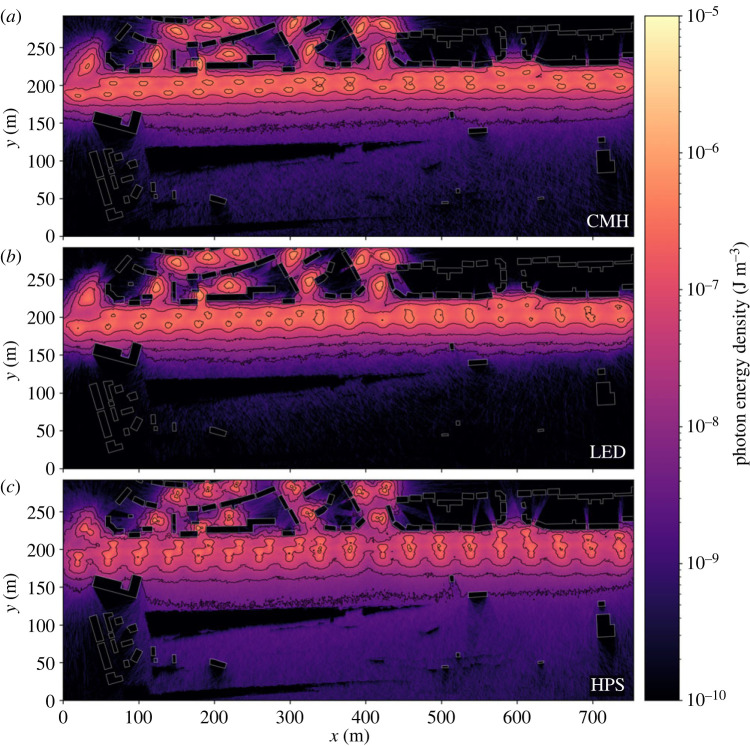
The ground-level energy density output by our MCRT model for CMH (*a*), LED (*b*) and HPS (*c*) light sources are placed along the road with a 40 m separation. Buildings are outlined in white. We apply contours at log_10_ energy density from −8.5 to −6.0 in steps of 0.5 dex. Even at the largest lamp spacing for which we ran simulations, shown here, the comparatively lower output and more porous barrier offered by the HPS lights compared with the other two is apparent.

**Figure 8.**
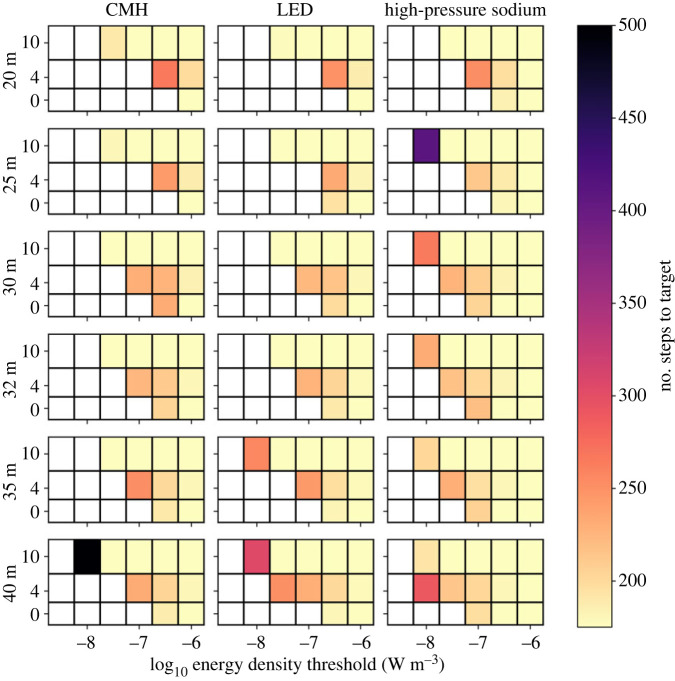
The success rate of the ensembles of random walkers for each scenario we constructed. The rows of the grid show spacing between light sources along the road increasing top-to-bottom. The three columns show results for CMH (left), LED (centre) and HPS (right) lamps. Each panel shows the number of steps successful walkers take to completely cross the simulation domain, at a given energy-density threshold (*x*-axis) and height above the ground (*y*-axis). This is intended as a measure of obstruction caused by the lighting environment, with larger numbers of steps representing a longer walk required to traverse the simulation. White cells indicate that no walkers successfully walked across the domain, suggesting a coherent light barrier across the domain.

Varying the light technology changes the distributions of successful walkers over a narrow range of thresholds, however, HPS lamps consistently allowed the largest number of walks to complete successfully in the fewest steps. This is evidenced by the HPS distributions (figure 6) exhibiting a narrower peak and a shorter tail than other light types for an equivalent scenario. We suggest that this results from two main factors. The radiant flux of the HPS light source is only around 60% that of the other two, for an equivalent input power (table 1), allowing more photophobic walkers to pass through the lit area. Furthermore, the lower uniformity HPS lamps create a relatively porous barrier, providing corridors of lesser illumination through which walkers may pass (figure 7). Increasing lamp spacing along the roadway proves effective at introducing darkness, with 40 m spacing offering corridors of minimum energy density 0.5 dex lower than 20 m spacing, through which photophobic walkers can more consistently pass (figure 8). Furthermore, the finer control over the directionality of emitted light offered by modern fixtures appears advantageous when the lights are placed at larger separations. This is especially true for walkers toward the lower energy threshold, which traverse an environment lit by LED lamps more effectively than one lit by CMH lamps (figure 6). While the radiant flux of both is comparable, the CMH lamps lack the discrete lens elements present on modern LED arrays.

**Figure 9.**
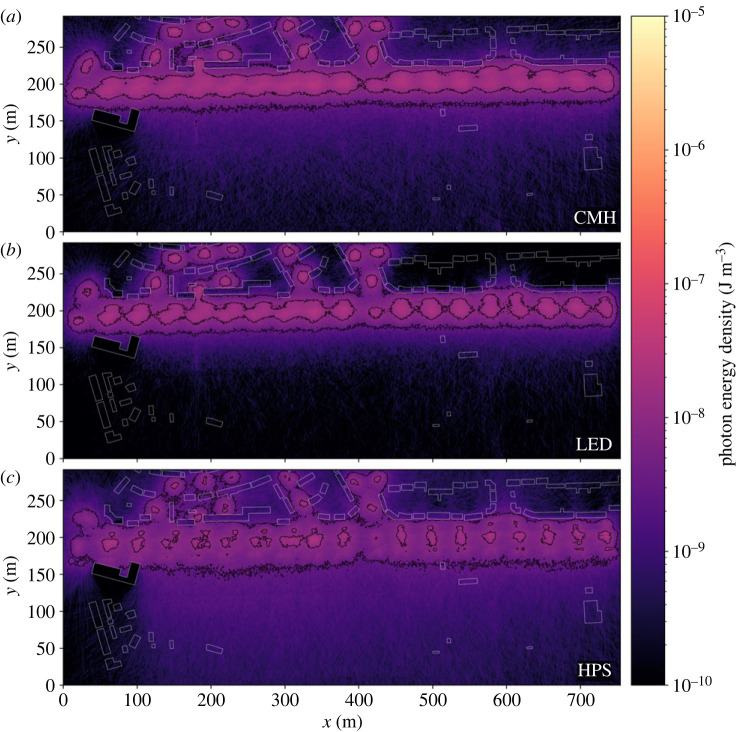
The energy density at 10 m above the ground output by our MCRT model for CMH (*a*), LED (*b*) and HPS (*c*) light sources placed along the road with a 40 m separation. Buildings are outlined in white. We apply contours at log_10_ energy density from −8.5 to −6.0 in steps of 0.5 dex. Note that the peak intensity of all lamps now appears comparable. The finer ability to focus light emission in LEDs has opened a corridor at around *x* = 420 that is not present in other simulations.

There is a strong correlation between walker success rate and height, with the lower threshold for successful walks being 1.0 dex lower at 10 m than at ground level (figure 6). Although modern lamp housings are designed to minimize direct upward emission, a fraction of their emitted light still upwells due to scattering and reflection in the environment. The asphalt material in this simulation consistently reflects around 10% of the incident light from all lamps across the entire visible spectrum. It is therefore interesting that the peak energy density for all the lamp types equalizes by 10 m above the ground (figure 9). We conclude that the modern lamps' higher uniformity is the cause, diluting the light over a larger area before reflection, whereas older HPS lamps will tend to pool their light closer to the light source, forming a concentrated column of illumination (figure 9).

**Figure 10.**
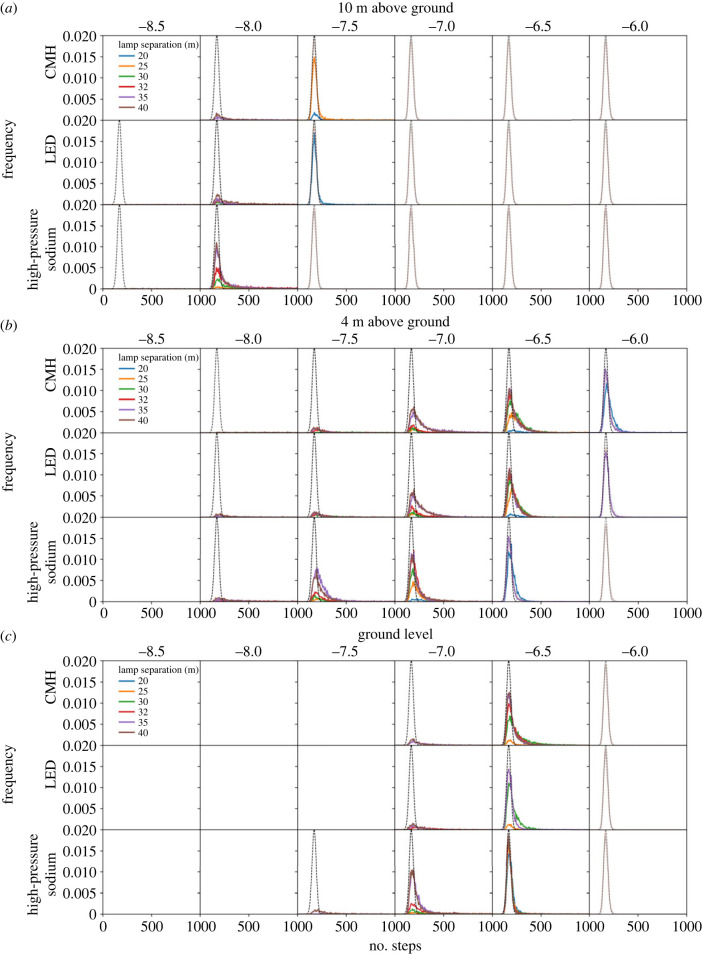
The distributions of random walker steps for the simulations for which we introduced an artificial dark corridor. Results are shown for planes at 10 m (*a*), 4 m (*b*) and 0 m (*c*) above ground level. The distributions are shown in the grids of panels as a function of representative lighting type (rows), log_10_ energy density threshold above which the proposed move is rejected (columns), and separation between lamps (colours). The nominal distribution is overlaid in each plot as a solid dashed line. No lines indicate no walks were successful. Those distributions for which all walkers are successful are shown faintly for reference. The dark corridor allows walkers with thresholds about 0.5 dex lower to successfully cross the domain.

Finally, we tested the effectiveness of dark corridors by running the simulation sweep with the deactivated streetlamp, as detailed in §2.3. On average, walkers with thresholds 0.5 dex lower were able to complete walks across the environment; however, there was an overall increase in successful walks of less than 1% (figure 10). By examining the successful heatmaps of walker visits, we were able to confirm that walkers starting from the position of the nearest lamp or closer were able to funnel through the dark corridor. Moreover, this dark corridor benefitted 3.1% of the walks from scenarios with some successful walks, bringing their trip length down by an average of 6.3%.

## 4. Discussion

Through even the simple simulations presented in this work, we have shown that our method can model the ground-level lighting distribution of real (figure 4) and hypothetical (figure 7) lighting environments. Moreover, as the MCRT method simulates the three-dimensional distribution of light, treating high-order effects, such as reflections and scattering, we can access the lighting environment above luminaires (figure 9). These initial simulations demonstrate that the issues surrounding the transition to new technologies, such as LED lamps, are complex, especially when viewed through an ecological lens. While the optics of modern lamps are more effective at preventing direct upward emission and light trespass into the environment, much care has been taken to design them with high uniformity, distributing their light evenly across the ground. Our simulations show that this effectively creates impenetrable light barriers along roads, reducing the availability of darkness and dark corridors within urban environments. Although considering only the near-field lighting environment, random walks demonstrate that introducing artificial dark corridors into environments are not necessarily a panacea to this issue. We therefore suggest that the entire visible lightscape should be considered holistically when designing lighting; considering how both animals and humans use, and traverse, them. A further worrying implication of the uniformity of modern lamps is their relatively high efficacy at distributing photons over the first order of reflection, widening the area over which reflected light is diluted. Understanding how this, combined with reduced trespass, shifts the balance between darkness and brightness within the environment, and the resulting effect upon skyglow, are vital for future studies of ALAN.

The generalizability of the MCRT method is one of the key strengths of our approach. Our model allows the physically accurate simulation of any lighting type for which the photometric data and spectra are available, in any real or hypothetical configuration. Owing to the model being three-dimensional, incorporating more thorough treatments of atmospheric scattering will allow us to probe the spectral and spatial extent of skyglow from first principles. With an appropriate treatment of attenuation of light due to the atmosphere, we can also make synthetic satellite observations of our simulated lighting environments—providing a strong connection to the vast archive of available observational remotely sensed datasets [56]. We are developing our tooling to increase the fidelity of generated digital twins. Our roadmap includes increases in detail for geometric meshes and materials, a foliage model to account for attenuation of light by trees and bushes, and a sky model for treatments of skyglow and diverse weather conditions. The MCRT method is also intrinsically parallel, in that the simulations of each photon packet are completely independent and can be run simultaneously. This property of our algorithm makes it highly scalable, hence we could perform analysis such as that presented in this work on much larger scales (e.g. city-scale) with similar fidelity. This is expedited by our pre-processing pipeline, which, given appropriate raster and vector data, can automatically generate a digital twin of the desired environment. Our toolkit provides in-roads to assessing habitat connectivity and dark corridors, and the upwelling of light and impacts of skyglow at, and beyond, city-wide scales.

To support this work, we are further improving the random walking algorithm we use to better emulate how animals experience the simulated lighting environment. Recall that the rasterized energy density planes, through which the walkers traversed, represent panchromatic measurements of the near-field radiant flux within the environment; meaning the random walkers are insensitive to colour. Furthermore, the naive weighted walking method favours forward progress, tending to get walkers stuck in concave areas of darkness, such as that between light pools. Hence, we suggest that dark corridors may be more beneficial than simulations predict, as real animals consider the whole visible lighting environment. By accounting for the field-of-view and spectral characteristics of animal vision systems in simulation outputs, as well as incorporating more realistic treatments of animal behaviour, we may better understand how animals perceive and use lighting environments.

We have demonstrated that our modelling methodology is robust, physically accurate and generalizable. By building upon the foundation presented in this work, there is the potential to develop a truly mechanistic method of predicting the spatial, spectral and temporal variations of ALAN. Our method addresses the resolution limitations of Earth observation data, and the cost of dedicated fieldwork campaigns, while providing access to the full three-dimensional lighting distribution and modelling of hypothetical scenarios.

## 5. Conclusion

In this work, we introduce our MCRT solver and its application to studying the ecological implications of ALAN. We have demonstrated our pipeline for creating digital twins, constructing, as an exemplar, a simulation of the Stoke Hill area of Exeter, UK. We created two-dimensional planes from the simulated three-dimensional energy density field and traversed them with weighted random walkers to assess how different streetlighting scenarios affect the availability of darkness within the studied environment.

The comparatively lower radiant flux of HPS lamps emits less light into the environment, but with high trespass. Their optics spread the light less evenly, creating a porous barrier of illumination through which organisms may pass more easily in all scenarios. Conversely, the optics of modern LED lamps allow them to distribute their output light more evenly on the ground with less trespass. Their high uniformity dilutes reflected energy density over a larger area, reaching parity with HPS lamps at 10 m above the ground. Crucially, this work demonstrates that considering the ecological impact of lighting design is complicated, with no single type of light providing an unmitigated improvement. Hence, we suggest that when designing lighting the entire visible lightscape should be considered holistically, accounting for how both animals and humans perceive and use it.

Given the success of these initial simulations, we have laid out a framework for developing the model presented in this work into a truly mechanistic method for studying ALAN.

## Data Availability

The model source code and input files from which the results presented in this publication were produced are available from the EIDC repository: https://doi.org/10.5285/1b64b008-8c20-4dd4-bf54-bf1894767a56 [57]. Supplementary material is available online [58].
